# Cohesive and Non Cohesive Golds—Correspondence

**Published:** 1881-03

**Authors:** 


### ARTICLE VII.

*Correspondence.*

Mr. Editor.—We are taught that cohesive or annealed gold is really softer than any other, and that though it may seem harder and harsher, it is made so by the instrument, the explanation being that the molecules being more cohesive, slip by one another less readily and so offer more resistance to alteration of shape or position. Now will you please tell me in what particular this differs from the property that produces hardness in any other substance, physically I mean, of course. Is it not this same cohesion of molecules and their resistance to displacement, which makes iron or ice hard? If it is this same cohesion of particles that makes iron harder to break or bend or compress than dough, this holding on of molecules that will not allow them to slip by, away from, or on to one another; how is it that it makes one thing hard and another soft? If it is meant that the individual molecule is softer, I have nothing to say about it, till some of our smart brethren devise a plan by which we can get hold of these uncertain little individuals so as to allow us to pack them into a cavity, one by one, and thus utilize their softness. Until then, as we will have to deal with them in the aggregate, and if as such they work stiffer and less yielding, would it not be as well to call such gold hard for the sake of simplicity?

Very respectfully,

J. W. F.

---

Perhaps, if we would for convenience sake call annealed gold adjustable foil, and the variety known as cohesive foil unadjustable, these would satisfy us just as well as any other terms. All pure gold with clean surfaces is cohesive; any which is non-cohesive is such because of slight alloying, or because of surface contamination. The alloy may be so slight as not to interfere with its purity practically.

It is understood, we believe, that Abbey's old-fashioned soft foil, than which there is none better, is alloyed with a trace of iron. Condensation of gases on the surface of foil interferes with its cohesiveness, many metals having the property of condensing gases on their surfaces,—possibly indeed a penetration of the gases into inter-molecular spaces. It may be worth while for our correspondent to keep in mind the fact that the most careful cleansing does not seem to make some of our purest metals, as platinum for example, cohesive, except at a white heat. We can only take the facts as they are, and sometimes act without explanation of them. Ice only coheres, we believe, by previous melting of the surfaces in contact. There is no explanation of why osmium for instance is infusible, or why iridium is so hard in its normal state; or why gold is soft, or tin easily fused. These are ultimate facts. Cohesive, or pure annealed foil *is softer* than that which is unannealed, just as truly as annealed gold plate is softer than that which has been hammered. But our dental nomenclature is yet new and as a consequence not exact. It is only a few years since cohesive foil was called *adhesive.* To come back to where we started, the old fashioned foil of Abbey's manufacture was eminently adjustable in the cavity, and so filled a place which the average operator finds it difficult to supply with other foils. The mallet is a good thing doubtless, but not the only good thing in dentistry; and evidently from the written and spoken opinions of many of the strictly cohesive foil operators, they have not the slightest idea of the capabilities of non-cohesive foil, or perhaps never saw an operation from a first class dentist in that style of work.

